# Neuropsychological Alterations in Patients with Congenital Hypothyroidism Treated with Levothyroxine: Linked Factors and Thyroid Hormone Hyposensitivity

**DOI:** 10.3390/jcm11123427

**Published:** 2022-06-15

**Authors:** Karla Cristina Razón-Hernández, Norma Osnaya-Brizuela, Armando Valenzuela-Peraza, Esperanza Ontiveros-Mendoza, Luis Miguel Rodríguez-Serrano, Jorge Pacheco-Rosado, Gerardo Barragán-Mejía, Karla Sánchez-Huerta

**Affiliations:** 1 Laboratorio de Neurociencias, Subdirección de Medicina Experimental, Instituto Nacional de Pediatría, Ciudad de Mexico 04530, Mexico; karcrisrazon@gmail.com (K.C.R.-H.); osnayanorma@hotmail.com (N.O.-B.); valenzuela.peraza2013@hotmail.com (A.V.-P.); 2 Laboratorio de Neurometría, Facultad de Estudios Superiores Iztacala, Universidad Nacional Autónoma de México, Ciudad de Mexico 04360, Mexico; 3 Centro de Investigación del Neurodesarrollo, Subdirección de Investigación Médica, Instituto Nacional de Pediatría, Ciudad de Mexico 04530, Mexico; espeonti@hotmail.com; 4 Laboratorio de Neurobiología de la Alimentación, Facultad de Estudios Superiores Iztacala, Universidad Nacional Autónoma de México, Tlalnepantla 54090, Mexico; cosmonauta84@yahoo.com.mx; 5 Departamento de Fisiología “Mauricio Russek”, Escuela Nacional de Ciencias Biológicas, Instituto Politécnico Nacional, Ciudad de Mexico 07738, Mexico; jpachecor@ipn.mx; 6 Laboratorio de Bacteriología Experimental, Subdirección de Medicina Experimental, Instituto Nacional de Pediatría, Ciudad de Mexico 04530, Mexico

**Keywords:** IQ, language, learning, memory, sensorimotor, visuospatial, visuoconstructive, infants, preschoolers, school children, adolescents, young adults

## Abstract

Eighty-five percent of the studies of patients with congenital hypothyroidism (CH) treated with Levothyroxine (L-T_4_) report neuropsychological sequelae throughout life. In neonates and infants, there is a deficit in sensorimotor skills (impaired balance). In preschool and elementary school children and adolescents, there are alterations in intellectual quotient (low scores), language (delayed phonological acquisition), memory (visual, verbal, visuospatial, visuoconstructive, autobiographical, and semantic), sensorimotor skills (impaired fine and gross motor control), and visuoconstructive–visuospatial domain (low scores in spatial location, block design, and object assembly). These neuropsychological domains are also affected in young adults, except for language (adequate verbal fluency) and visuoconstructive–visuospatial domain (no data). The onset and severity of neuropsychological sequelae in patients with treated CH depend on several factors: extrinsic, related to L-T_4_ treatment and social aspects, and intrinsic, such as severity and etiology of CH, as well as structural and physiological changes in the brain. In this review, we hypothesized that thyroid hormone hyposensitivity (THH) could also contribute to neuropsychological alterations by reducing the effectiveness of L-T_4_ treatment in the brain. Thus, further research could approach the THH hypothesis at basic and clinical levels to implement new endocrinological and neuropsychological therapies for CH patients.

## 1. Introduction

Congenital hypothyroidism (CH) is an endocrine disorder characterized by a deficiency of thyroid hormones (THs) at birth. This pathology is mainly caused by abnormalities in the thyroid gland’s development (dysgenesis) or by alterations in THs biosynthesis (dyshormonogenesis) during the embryonic stage [1]. CH represents the most frequent disorder of the endocrine system in newborns, with a prevalence of 1 in 2000–3000 newborns [2] and a predominance of 2:1 in females [3]. In Mexico, the prevalence of the CH was of 1 in 2426 newborns in 2004 [4]. As in other countries [5,6], the prevalence has increased over time, bringing the national rate to 1 in 1373 newborns in 2018 [7].

CH is diagnosed by neonatal screening, an essential tool for the detection of CH and other metabolic and congenital diseases [8]. The neonatal screening quantifies the concentrations of thyroxine (T_4_) and thyrotropin (TSH) in a blood sample collected a few days after birth. Elevated TSH levels together with low levels of free T_4_ (FT_4_), compared with the reference values specific for the infant’s age, confirm the diagnosis of CH [2,9]. According to Therrell et al. [10], the detection of CH has spread worldwide: North America (USA and Canada) and Europe (47 of 48 countries) have the highest rate of neonatal screening implementation, followed by Latin America (16 of 20 countries). In the Middle East and North Africa, screenings are applied in 15 of 21 countries, while 13 of 23 countries in the Asia–Pacific region carry them out. Although CH detection has progressed greatly worldwide, there are still countries with a low coverage: Bolivia (~20%), Peru (~20%), Guatemala (~1%), and the Dominican Republic (~1%) in Latin America; Iraq (20%) and Lebanon (50%) in the Middle East and North Africa; and Sri Lanka (27%), Bangladesh (<5%), Cambodia (<3%), Indonesia (<2%), India (<1%), Pakistan (<1%), and Vietnam (1%) in the Asia–Pacific region. In Mexico, the coverage of the neonatal screening is around 80% [10].

Levothyroxine (L-T_4_) monotherapy is the treatment of choice for CH at an initial dose of 10–15 µg/kg/day for infants with severe CH (fT_4_ < 5 pmol/L). A lower dose (~10 µg/kg/day) is used for the treatment of mild cases. L-T_4_ treatment should be started as soon as possible, no later than two weeks after birth (early treatment) [2,11]. Based on the above criteria, L-T_4_ treatment is optimum when patients receive both the appropriate dose of L-T_4_ and early treatment.

Untreated CH leads to intellectual disability, dwarfism, ossification defects, and deafness [12]. Currently, neonatal screening allows early diagnosis and treatment of CH, thus reducing hypothyroidism in the infant’s brain and the risk of suffering from intellectual disability. However, with exceptions [13,14,15,16,17,18,19,20,21,22], the neurodevelopment follow-up shows subtle sequelae in children with CH treated with L-T_4_ (CH-Tx) [23,24,25,26,27,28], an issue that we discuss extensively in this review. Many of these alterations have been reported in patients with a non-optimum L-T_4_ treatment (references in Table S1B), and some of them are from patients treated with suitable doses of L-T_4_ and early treatment (Table S1A) [29,30,31,32,33,34,35,36]. This work compiles the neuropsychological alterations reported in patients with CH-Tx, from neonates and infants to young adults, with emphasis on the main neuropsychological domains affected. This review includes a comprehensive analysis of the main factors contributing to the onset of neuropsychological sequelae in patients with CH-Tx. Additionally, we hypothesize that hyposensitivity to THs also contributes to these neuropsychological alterations. Finally, a research agenda aimed at contributing to this area of study is provided.

## 2. Methods

PubMed, Scientific Electronic Library Online (SciELO), and Google Scholar databases were used in the literature review. Scientific articles in English and Spanish were included with no restrictions regarding publication dates.

The following keywords were used in the neuropsychology section: primary congenital hypothyroidism, neuropsychological profile, neurodevelopment, levothyroxine, IQ, language, speech disorders, communication, learning, memory, sensorimotor domain, psychomotor development, and visuospatial and visuoconstructive abilities. Clinical reports on neuropsychological profiles of patients with other types of hypothyroidism (endemic cretinism, central congenital hypothyroidism, subclinical hypothyroidism, maternal hypothyroxinemia, hypothyroidism due to THs resistance, Allan–Herndon–Dudley syndrome, or any type of acquired hypothyroidism) were excluded.

In the section on associated factors, the following keywords were used: congenital hypothyroidism, initiation of L-T_4_ treatment, dose of L-T_4_, socioeconomic status, primary caregivers, severe congenital hypothyroidism, athyreosis, ectopia, dyshormonogenesis, magnetic resonance imaging, electroencephalogram, and thyroid hormones hyposensitivity.

## 3. Neuropsychological Alterations in CH-Tx

Neuropsychology applies standardized psychometric tests to examine the relationship between brain functioning and higher cognitive processes [37]. These psychometric tests evaluate different neuropsychological domains such as language (ability to perform symbolic communication), learning (ability to acquire new information), memory (ability to retain previously learned information), motor functioning (fine and gross motor skills), visuospatial and visuoconstructive skills (ability to represent, analyze, and manipulate objects mentally), and intellectual quotient (IQ, general cognitive ability of neuropsychological domains) [38].

**Figure 1:**
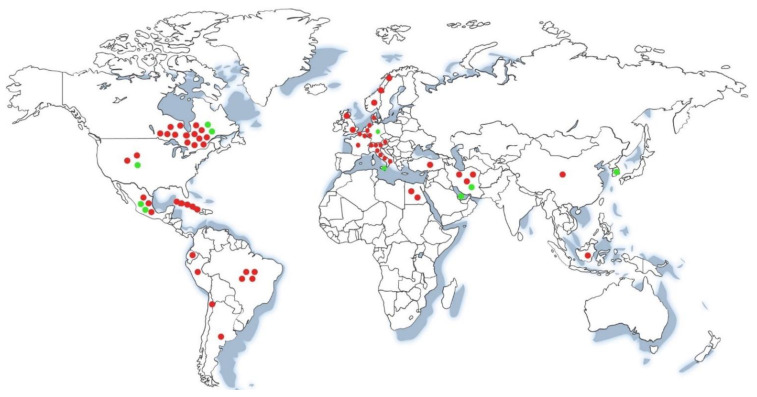
**Worldwide distribution of scientific reports on neuropsychological profiles of CH-Tx patients.** The red dots indicate reports of CH-Tx patients with a deficit in at least one neuropsychological domain. The green dots indicate reports of CH-Tx patients who did not present neuropsychological alterations.

Several psychometric tests have been used worldwide to assess the cognitive capacity of patients with CH-Tx (Table S2) leading to the publication of scientific reports. The largest number of reports come from North America (34.8%), followed by Europe (30.4%), Latin America (18.8%), Asia (13.0%), and Africa (2.9%). These data agree with the coverage of neonatal screening in the different regions [10]. Interestingly, 85.5% of the studies (59/69) analyzed in this review reported a deficit in at least one neuropsychological domain in patients with CH-Tx, while 14.5% (10/69) of the studies showed that CH-Tx patients did not present neuropsychological alterations (Figure 1 and Table S1). It should be noted that 13.0% (9/69) of the total of reviewed articles reported evidence from patients with an optimum L-T_4_ treatment, 50.7% (35/69) showed evidence from patients without optimum treatment, and 36.2% (25/69) were articles with imprecise data or no data of L-T_4_ treatment (Table S1).

This review includes a broad description of the neuropsychological alterations reported in CH-Tx patients, considering six neuropsychological domains (IQ, language, learning, memory, sensorimotor domain, and visuospatial and visuoconstructive abilities) and five age groups: neonates and infants (0–2 years), preschoolers (3–6 years), elementary school children (7–12 years), adolescents (13–18 years), and young adults (19–30 years).

### 3.1. IQ

In the 1970s, the relationship between IQ deficiency and CH in children began to be studied [39]. It has been shown that the cognitive development in children with CH depends on the early diagnosis and optimum treatment, which should be monitored during the first years of life [40]. This is supported by the analysis carried out in Table S3II, which shows that 100% (8/8) of the papers reporting optimum L-T_4_ treatment describe full-scale IQ scores within the reference values (IQ score: 90–109) [18,29,30,32,33,34,35,36].

On the other hand, several studies have showed deficits in IQ scores in patients of different ages with CH-Tx. At preschool age, the mean of the full-scale IQ score of CH-Tx patients, who initiated treatment at postnatal day 16–18 [41] or at less than one month of age [42], is clinically lower than normative data, and this IQ-score correlates with serum T_4_ levels at two years of life [43]. Clinical follow-up studies conducted at elementary school age highlight that children with CH-Tx have lower verbal, performance, and full-scale IQ scores [44,45,46,47] when treatment is initiated at postnatal day 23 [44]. These data are consistent with reports indicating that these children show the lowest IQ scores in vocabulary and overall performance [48]. In adolescence (13 years and older) and early adulthood (21.5 years), the follow-up studies showed that CH-Tx patients had lower IQ scores on the verbal, full-scale, and performance scales than established in normative data. This indicates that the sequelae are maintained as patients grow older when the L-T_4_ treatment is initiated late (between 13 and 27 days of life) [36,48,49].

Factors linked to L-T_4_ treatment play a crucial role in the IQ score obtained by CH patients. The dose of L-T_4_ used to maintain a euthyroid state in the first years of life is a key factor in achieving an adequate IQ, as the administration of lower doses are associated with reduced IQ in children at 6 years of age [29]. The time-to-treatment initiation with L-T_4_ is also essential to limit alterations in intellectual development: 78% of CH infants treated before 3 months of age showed higher IQ scores (IQ = 89) compared with those treated after 7 months of age (IQ = 54) [50]. In CH-Tx children evaluated between 4 and 14 years of age, the IQ scores were lower when the treatment began after 30 days of age [22,51,52,53] compared with patients with early treatment. CH-Tx patients evaluated between the ages of 6–21 years, whose time-to-treatment initiation is around postnatal day 29, also show low IQ scores [49,52]. Follow-up studies during puberty and young adulthood (CH-Tx patients aged 13 years or older with suboptimal treatment) showed that patients had lower IQ scores on the verbal, full-scale, and performance scales than did the normative population [54]. In addition to factors linked L-T_4_ treatment, evidence shows that a lower IQ score is correlated with a more severe initial hypothyroid state [14,19,55,56] and with low T_4_ levels during the first years of life [56].

In contrast, other works have reported that time-to-treatment initiation between 7 and 60 days of age prevents the deterioration of IQ scores in children aged 2–7 years [15,18,19,20,21,29], 8–10 [22,51] and adolescents at age 12 [57]. Some reports describe an improvement in IQ at 6 years of age, although a regular score is not fully reached [56]. Other authors point out that even when the IQ scores of children with CH-Tx are within normal limits, they have lower IQ values than healthy controls at ages 5 [42], 9 [58], and 11 to 15 [17,30,57,59,60,61]. Bongers-Schokking et al. also reported CH-Tx patients with suboptimal treatment and with abnormalities in visuomotor and verbal scores, but normal IQ scores [29].

All studies agree that the time-to-treatment initiation is essential to limit the damage to cognitive development. It is clear that a decrease in IQ scores is observed if CH is detected months after birth and the treatment starts late [62]. The optimum time of L-T_4_ treatment remains a matter of debate. Herrera-Chinchay et al. recently reported 21 days of age as a critical time: they mention that children who start treatment before 21 days of age have no IQ deficits, while 35% of those who start after 21 days have mild or moderate deficits [63]. In contrast, Bargagna et al. reported that children with CH who started treatment between 15 and 45 days of age show no deficits in IQ measured at 3, 5, and 7 years of age [64].

The dose of L-T_4_ used in children with CH is another crucial factor that must be also considered for best outcomes. Studies have compared three initial doses of L-T_4_, demonstrating that thyroid function must be normalized as soon as possible to prevent a decrease in IQ, as measured at ages 3–8 years [34,65]. The adequate treatment should not exceed the dose of L-T_4_. Overdosing is not beneficial, as reported by Bongers-Schokking et al., who found lower IQ scores in overdosed children aged 1–11 years [66,67]. Interestingly, it is suggested that although the treatment with L-T_4_ restores IQ, the level of sustained attention is lower in children with CH aged 7–13 years [56]. García Morales et al. showed that patients with CH have attention deficits at age 9 years when a high dose of L-T_4_ is administered during the first years of life [31]. This attention deficit is not accompanied by hyperactivity, so it is difficult to identify.

### 3.2. Language

Language development is also altered in children with CH-Tx. Several reports show the presence of phonological deficits and significant delays in the development of expressive and receptive language in infants [68,69,70,71]; this is even clearer in children whose hypothyroidism is not properly controlled [72]. The scores obtained show that children with CH-Tx, specifically those aged 6 and 7 years, have a delay in phonological acquisition compared with the control groups [69]. Cooper et al. demonstrated that school children and adolescents (age 6–15 years) with CH-Tx have lower communication capacity than controls, although within normal limits [52]. Studies that evaluated linguistic competencies in children with CH-Tx during the preschool and school stages (5–8 years and 9–12 years, respectively) showed that members of the younger group achieved average scores in that domain. In contrast, those in the older age group showed a moderate delay in expressive and receptive language, especially in tasks of sentence and oral instruction comprehension, a fact that conditions their academic performance in school [54,73]. Bargagna et al. reported that only 50% of the 24 children with CH who began treatment with L-T_4_ at 15–45 days of age showed normal linguistic development, while 29% presented phonological disorder, and 21%, morphosyntactic disorder at 3 years of age. These figures were reduced when the evaluation was carried out when the children were 7 years of age, thus 71% of them showed no linguistic alterations and 29% presented a phonological disorder [64]. The results agree with those reported by Gejao et al., who showed that approximately 23% of children with CH-Tx have phonetic problems at 3 years of age [74].

The studies suggest that language deficits in children with CH-Tx have a multifactorial origin. The ability to generate fine movements is essential, since it has been reported that children with CH-Tx who present fine movement abnormalities have twice the language deficits as those who do not [75]. Another factor in language deficit is memory, given that the tests used for linguistic assessment involve the repetition of orders. Then, responses may be impeded not so much by a lack of understanding but by memory impairment [76]. Likewise, language deficits become evident with age due to the increase in test complexity [73]. As highlighted, the time-to-treatment initiation is relevant: a delay in the onset of language has been reported in 90% of children who start thyroid treatment after 21 days of age [63], which emphasizes the need for an early detection and treatment of CH.

It must be noted that there is a correlation between language development (assessed from 3 to 18 months of age) and T_4_ levels detected at birth [51]. This can be interpreted as the inability to complete neuronal maturation due to hormonal deficiency, as children with language deficiencies also show signs of immaturity in the electroencephalogram [77].

### 3.3. Learning

Memory impairment [78] and sustained attention deficit [79] in preschool children with CH can affect the learning process. Patients with CH-Tx evaluated during the school period presented alterations in several neuropsychological tasks linked to different types of learning. The score obtained in mathematical skills was significantly lower in children who presented T_4_ values ≤ 40 nmol/L prior to treatment than in those who presented T_4_ values > 40 nmol/L [80]. It has also been reported that CH-Tx children have a lower performance in mathematics at the beginning of their education but reach scores similar to those of the rest of the population by sixth grade [54]. The above consistently shows that children exhibit adaptive difficulties within the school environment.

Oerbeck et al. evaluated young adults (20 years of age) with detected and treated CH around 24 days of age. They showed no alterations in verbal fluency but some in mathematical skills. Furthermore, 24% of adults with CH-Tx and 6% of the control group who started high school did not graduate [81]. These results agree with those of Rochiccioli et al., who reported that children with CH-Tx have lower performance in mathematics, evaluated in primary and secondary education. In addition, they found that more children with CH repeated first grade in elementary school compared with the control group [80].

In contrast, Bargagna et al. reported that 21% of children with CH-Tx evaluated between 5 and 10 years of age presented learning difficulties, more evident in the writing and copying of figures, but no alterations in mathematics [82].

### 3.4. Memory

Memory is another domain affected in patients with CH-Tx, which is consistent with the multiple morphological and functional alterations induced by perinatal hypothyroidism in the hippocampus, a brain region widely related to learning processes and memory [83,84]. According to studies, children with CH-Tx present scores significantly below than their peers on the memory subtest of the Wechsler scale. Children with CH-Tx are outperformed by controls and have a greater deficit in the processing of long-term semantic memory during the school stage (9–10 years), which can be related to structural and functional changes in the hippocampus [58]. Research shows that memory tasks, in which children aged 7–8 years are required to recall an event or remember a task, are the most sensitive to the effects of early and subsequent doses [33]. It has also been shown that patients with CH-Tx aged between 11 and 15 years present a deficit in associative visuospatial memory tasks [16], a poor index of verbal memory and memorization, and atypical hippocampal activity [36,54,61]. Hepworth et al. evaluated patients with CH-Tx aged 11–14 years in word recognition memory tasks, and their electrophysiological response by event-related potentials (ERPs). Interestingly, deficits were found in the electrophysiological response in temporal regions, which is related to an abnormal hippocampal development [17].

Consistently, Weber et al. showed the existence of deficits in visuoconstructive memory tasks in CH-Tx patients aged 6–10 years [60]. Moreover, deficits in the visual memory index have been reported in CH patients evaluated at the ages of 7–12 years. Their TSH levels were normalized after 2 months of age when compared with children treated early [85]. Additionally, hypothyroid patients aged 9–14 years and treated with L-T_4_ present a compromised episodic memory while the semantic one remains unchanged. They show lower precision in providing details of events, which is related to the presence of smaller hippocampal volumes [24,36,86].

In young adults (20 years), Oerbeck et al. found that CH-Tx is associated with a deficit in visual and verbal memory. They suggest that an initial dose of L-T_4_ ≥ 7.8 µg/kg/day might improve the performance of these types of memory [87].

### 3.5. Sensorimotor Domain

The sensorimotor is one of the most affected domains of the neuropsychological functioning of children with CH-Tx who show deficits in fine and gross motor skills. Descriptive studies conducted during the preschool stage have highlighted that global fine and gross motor functioning is commonly affected in children with CH-Tx [70].

Within this domain, children evaluated at 3, 5, and 7 years of age consistently show a slower reaction, poorer visual–motor accuracy [64,68,75], and motor deficits at an average age of 10 years [55]. When evaluated from 6 months to 4 years of age, those with treated CH show difficulties in balance and motor dexterity speed. This is anatomically correlated with a complex interaction of central processing, which mainly involves brain structures such as the motor cortex, basal ganglia, cerebellum, and vestibular system [88]. Additionally, studies conducted in adolescents show that patients with CH-Tx manifest deficits in the fine motor area at the age of 13–14 years [54], while young adults (21 years) with severe CH presented motor difficulties related to balance as compared with control groups [49].

Some studies suggest that motor skills performance is highly dependent on T_4_ values prior to treatment. This is because a reduction in the scores of motor coordination and processing speed can be observed in children with CH who presented T_4_ levels ≤ 40 nmol/L vs children who showed levels ≥ 40 nmol/L and healthy controls during school age [84]. Similarly, Corral-Guillé et al. showed that patients with CH aged 4–6 years who received treatment with L-T_4_ showed an improvement in their motor functions [89].

### 3.6. Visuospatial and Visuoconstructive Skills

It has been found that visuospatial deficits are frequently observed in children with CH-Tx during preschool age because, within this domain, they have low scores in the tasks of spatial placement and execution [78]. Consistently, during the school period, children with CH-Tx still perform poorly in visuospatial tests, specifically in design tasks with cubes and object composition measured by the Wechsler scale [58]. In addition, it has been reported that CH-Tx patients aged 8–10 years show less activation of the parietal cortex, which is accompanied by difficulty in detecting and processing visual stimuli [59]. Follow-up studies show that during adolescence, patients with CH treated with L-T_4_ show poor performance in visuoperceptual (processing of spatial relationships between objects), visuospatial (geometric puzzles), and visuoconstructive tasks (design with cubes) when compared with the control group [35,54,90]. Although the cause of the deficits in this domain is unknown, the literature suggests that they are anatomically related to a delay in the maturation of the magnocellular system. The latter originates anatomically in the retina and communicates with diencephalic structures (thalamus), projecting information toward the occipito-parietal cortex, related to movement direction and the location of objects in space [91].

### 3.7. Optimum Neuropsychological Domains in Patients with CH-Tx

After analyzing the literature on the neuropsychological profile of patients with CH-Tx, we found evidence supporting that these patients have an optimum performance in specific neuropsychological domains. Concerning IQ scores, we found that 85.7% (42/49) of the papers report CH-Tx patients with full-scale IQ scores within the reference values (Table S3I). Moreover, 53.6% (15/28) of the case–control studies show that CH-Tx patients have full-scale IQ scores similar to those obtained by controls (Table S3I). Finally, Arreola-Ramírez et al. reported that 75% of the patients with CH-Tx showed a normal IQ score, while 8.3% exhibited elevated IQ scores [15]. With regard to memory, other authors have shown that school-age children and adolescents with CH-Tx have satisfactory scores in visuospatial associative and short-term recognition memory [16,17].

To clarify whether satisfactory neuropsychological outcomes are associated with optimum L-T_4_ treatment, we analyzed the full-scale IQ scores of CH patients who received optimum treatment. Interesting, we found that all studies of CH patients with optimum L-T_4_ treatment reported full-scale IQ scores within reference values (Table S3II) [18,29,30,32,33,34,35,36]. On the other hand, when the analysis is restricted to case–control studies [29,30,32,34,35,36], we found that 50% (3/6) of the articles showed full-scale IQ scores similar to those obtained by control children [29,32,34], while the other half had low full-scale IQ scores [30,35,36]. This analysis provides evidence for the importance of the optimum L-T_4_ treatment by supporting that both early treatment and adequate L-T_4_ doses result in IQ scores within the normal range and prevent CH patients from obtaining scores classified as low average, borderline, or extremely low. Importantly, we only found and analyzed eight papers that reported optimum treatment (based on the criteria in [2]), thus highlighting the importance of further research on optimally treated CH patients.

### 3.8. Summary of Neuropsychological Alterations in Patients with CH-Tx by Age Group

**Figure 2:**
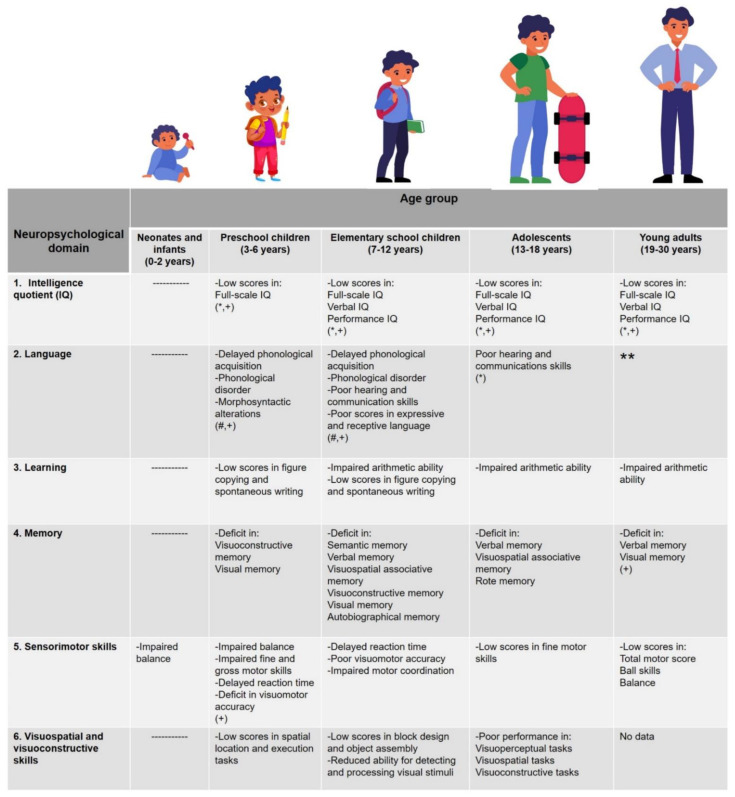
**Main neuropsychological alterations reported in CH-Tx patients at different ages.** This figure summarizes the main alterations reported in CH-Tx patients; however, these alterations depend on several factors (see Section 4). The dotted line indicates that it is not possible to evaluate the neuropsychological domain in neonates and infants. * Scores are frequently within normal limits, but values are at inferior limit. + Optimum L-T_4_ dose and early treatment are essential for better scores. # Alterations were found in 29–50% of children with CH. ** No impairment in verbal fluency.

Figure 2 shows the main neuropsychological alterations found in patients with CH-Tx in five age groups: neonates and infants, preschoolers, elementary school children, adolescents, and young adults. In the neonatal stage and childhood, alterations occur in the sensorimotor domain. In preschool, school, and adolescence, the six neuropsychological domains analyzed are affected, with a greater number of alterations reported in school age. In early adulthood, language seems to reach optimum levels, although IQ, mathematical skills, memory, and sensorimotor domains still show a deficit. However, the conclusions for this last age group should be taken with caution, as reports are scarce. It should be noted that most of the neuropsychological alterations are mild; some of them can be mitigated by an optimum dose of L-T_4_ and early treatment, and not all of them are present in the same individual at specific ages.

The information contained in this figure could constitute a relevant clinical guide for pediatricians, endocrinologists, psychologists, and other health professionals who face the challenge of characterizing the neuropsychological profile of CH patients at different ages. This guide will allow for a faster diagnosis of neuropsychological alterations, promoting the early incorporation of patients into therapeutic intervention programs.

## 4. Factors Contributing to the Establishment of Neuropsychological Sequelae in Patients with CH

Clinical evidence on the neuropsychological performance of patients with CH reveals that, despite treatment with L-T_4_, these individuals present a significant deficit in different domains of cognitive, sensory, and motor function (see Section 3). To date, the cause of this deficit is not fully clear yet; however, clinical findings suggest a multifactorial panorama that may contribute to the establishment of neuropsychological sequelae in these patients.

**Figure 3:**
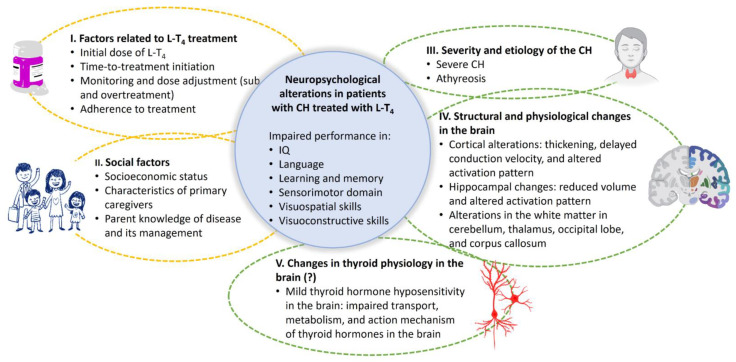
**Factors related to the neuropsychological alterations found in patients with congenital hypothyroidism treated with levothyroxine (L-T_4_).** The onset and severity of the neuropsychological alterations in patients with congenital hypothyroidism treated with L-T_4_ are related to extrinsic (dotted yellow line) and intrinsic (dotted green line) factors. Extrinsic factors include those related to L-T_4_ treatment (I) and social elements (II). Intrinsic factors correspond to severity and etiology of congenital hypothyroidism (III), and changes in the brain, such as structural and physiological alterations in specific brain regions (IV) and modifications in the thyroid physiology of the brain (V). This review proposes the hypothesis that modifications in the brain thyroid physiology lead to a state of mild thyroid hormone hyposensitivity that could also contribute to the onset of the neuropsychological sequelae (V).

This panorama can include extrinsic and intrinsic factors. Among the first, are social factors (socioeconomic status, family environment, and characteristics of primary caregivers) and those linked to treatment with L-T_4_ (time-to-treatment initiation, initial dosage, monitoring and dose adjustment, and adherence to treatment). On the other hand, intrinsic factors refer to patient-specific factors. They can include the etiology and severity of CH, anatomical and physiological changes in specific brain regions, and possible molecular changes in cerebral thyroid physiology. The last two are likely induced by THs deficiency during late pregnancy and/or the first days of extrauterine life (Figure 3).

### 4.1. Factors Linked to Treatment with L-T_4_

The factors linked to treatment with L-T_4_ are determinants for the evolution and limitation of the sequelae in patients diagnosed with CH. According to recommendations, the treatment must be started immediately, preferably within the first two weeks of life, with an initial dosage of 10–15 μg/kg/day of L-T_4_ [2,92]. Maintaining serum TSH, FT_4_, and T_4_ within the age-specific reference range is ideal [93]. It has been shown that early time-to-treatment initiation, with L-T_4_ doses of ~11.4 μg/kg/day, favors the development and integral growth of children with CH, who can achieve a performance comparable to that in healthy children [94].

The time-to-treatment initiation and the initial dose of L-T_4_ are the most studied factors due to their relevance in the onset of neuropsychological sequelae in patients with CH-Tx. Overall, the evidence shows that as time-to-treatment initiation is delayed and suboptimal doses of L-T_4_ are administered, children obtain lower IQ scores [18,21,22,29,34,50,57,63,64,65,89] and have delayed language development [63]. A deeper discussion of these two factors and their relationship with the neuropsychological sequelae of the patients is addressed in Section 3 of this review.

Constant monitoring and dosage adjustment are recommended to avoid the adverse effects of undertreatment or overdose [95]. An initial overtreatment (0–5 months of age) at dosages > 10 μg/kg/day has been associated with mean FT_4_ values of 26.8 ± 9.4 pmol/L. This results in a decrease in IQ scores when children are aged 2–11 years old and at high risk (5.5 times more) of obtaining an IQ < 85, a score that place them below normal values [67].The follow-up of CH patients reported that those treated with overdoses in early stages (1–3 months of age) obtained high scores associated with attention deficit and hyperactivity syndrome at 11 years of age [96]. This shows that behavioral disorders are also related to overdoses of L-T_4_.

As in any other chronic condition, adherence to L-T_4_ treatment is essential for best clinical outcomes in the CH. A study conducted in the USA by Kemper et al. reported that around 35% of children with CH discontinued their treatment at 3 years of age. Among them, half of the children dropped out of treatment after two years and the other half after three years. Some of the patients (29–47%) who discontinued the treatment before three years did not undergo a TSH test in the 180 days following the date of their last prescription [97].

Molecular biology has become an essential tool to study mechanisms related to chronic diseases. Works on genetics have revealed the contribution of multiple genes to the origin of CH: TSHR, FOXE1, NKX2-1, PAX8, and NKX2-5 have been implicated in thyroid dysgenesis; while TPO, DUOX2, TG, SLC5A5, SLC26A4, and IYD have been related to dyshormonogenesis. However, defects in iodothyronine transporters and resistance to THs have also been associated with genetic mutations [98,99].

To date, the administration of liothyronine (L-T_3_) in combination with L-T_4_ has been shown to be favorable for the normalization of TSH and T_4_, although it has only been used in children with central resistance to THs [100]. In view of the results of the CH treatment and its sequelae, studying the biochemical and genetic mechanisms underlying the response to the treatment is a promising path. This approach will help us to deepen our understanding of the pathophysiology of CH and contribute to the design of new therapeutic strategies for the treatment of this disorder.

### 4.2. Social Factors

The role of the family and the social environment is important in supporting the patient with CH. Less favorable neuropsychological results are associated with a poor socioeconomic environment. Several studies have shown that the socioeconomic status of the family affects cognitive development of CH patients. There is a positive correlation between the socioeconomic conditions of the family and the educational level of the mother with the development of the children’s language and executive functions. As the family’s income and the mother’s education level increase, the child obtains higher IQ scores [70,71,101]. Adolescents with a diagnosis of agenesis and low socioeconomic status have a higher risk of suffering an adverse outcome, showing a difference of up to 10 points in the IQ score [30]. The characteristics of the primary caregivers of CH patients are also of great relevance for the neuropsychological outcome. A study conducted in Brazil reported that women are the main caregivers (94.3%) of CH children. Over a third of the caregivers had little knowledge of the disease (37.3%), and approximately a quarter were unaware of the meaning of CH (24.1%). More than half of the study group (59.5%) was classified as working class, while 35.7% had not completed elementary school [101]. These data suggest that a high proportion of patients with CH live in disadvantaged environments with caregivers who have little knowledge of the disease, increasing the risk of neuropsychological sequelae, especially in IQ.

The enrollment of patients with CH in early intervention programs has been a useful strategy for the treatment and limitation of sequelae. Studies by Rivera et al. and Díaz-Pérez et al. describe the importance of the mother’s active participation, the stimulation received at home, and the incorporation to stimulation programs in the cognitive development of children [56,102]. Rivera et al. reported that children with CH, who had early neuropsychological intervention and hormone replacement treatment, scored better than healthy controls. This suggests that favoring mother-child interaction by their enrollment into an early intervention program is beneficial for the child’s intellectual development [102]. Pérez et al. highlighted that the mother’s participation, high stimulation at home, and a better socioeconomic status act as protective factors for child development [56].

### 4.3. Severity and Etiology of CH

Pioneering studies conducted in the early 1990s demonstrated that the severity and etiology (agenesis, ectopy, or dyshormonogenesis) of CH have a great impact on the neuropsychological performance of patients with CH [39,43,103].

The severity of hypothyroidism is determined by serum levels of T_4_ at the time of diagnosis (or initial T_4_); an initial T_4_ concentration lower than 2 µg/dL is considered an indicator of severe CH [34,49,60,64]. Overall, the evidence supports that severe CH is associated with deficits in IQ, language, and motor skills. Several authors have shown that patients with severe CH have lower scores in full-scale, verbal, and performance IQ than patients with moderate or mild CH as measured in preschool [65,103], school [34,55,60], and early adulthood [49]. A positive association has also been found between initial T_4_ levels and the score for full-scale or performance IQ [20,49,57]. Regarding language, there is a high frequency of language disorders in children with severe CH [64], and it is known that low initial T_4_ levels predict a poor performance in this neuropsychological domain [71,104]. Motor skills are also affected in severe CH: there are greater motor deficits and more severe alterations in balance, limb coordination, fine and gross motor skills, quality of movement, and manual dexterity compared with children with milder CH [29,49,64,71,103]. It is worth mentioning that the deficits associated with severe CH are partially reversed with early treatment and with optimal initial doses of L-T_4_ [29,65].

The etiology of CH is also a key factor in the neuropsychological outcome of patients with CH; agenesis is the main risk factor for presenting deficits of greater severity in IQ, language, and the sensorimotor and visuospatial domains. Thus, verbal, performance and full-scale IQ are lower in children with agenesis than in those with ectopia or dyshormonogenesis [43,57,85,89]. Díaz et al. [56] emphasized that the etiology of agenesis reduces the total IQ score by 11.6 points; in fact, there is a greater frequency of patients with an IQ that reaches mental deficiency, borderline, or normal clumsy. A greater deficit in speech and expressive and receptive language has been reported, along with a greater frequency of phonological deviation in patients with agenesis [39,69]. Regarding sensorimotor and visuospatial domains, patients with CH due to agenesis show deficient performance in eye-hand coordination, locomotion, and perceptual area compared with children with CH due to ectopia or dyshormonogenesis. Interestingly, the etiology of CH has no effect on memory [39].

The literature supports that severe CH and agenesis are important risk factors for developing some neuropsychological alteration to a greater degree. Both factors are postulated to be associated with very low concentrations of T_4_ during the last trimester of gestation and in the early postnatal stage, which could more severely affect the neurodevelopment of various brain regions that mature in this period.

### 4.4. Anatomical and Physiological Brain Changes in Patients with CH-Tx

Several works have evaluated the neuroanatomical and neurophysiological alterations in the brain of patients with CH-Tx. Using magnetic resonance imaging (MRI), school-age children and adolescents (10–14 years) with CH-Tx show an incidence of cerebral structural abnormalities similar to that of the normative population [105]. Although reports do not show structural abnormalities that lead to severe disabilities, multiple studies have described subtle neuroanatomical alterations that correlate with deficits in some neuropsychological domains.

An MRI study showed that school-age children and adolescents with CH-Tx (ages 9–16 years) exhibit changes in the thickness of certain brain cortex regions as compared with their controls. Specifically, a thinning is found in the cortical gyrus (left hemisphere: superior frontal gyrus and superior parietal gyrus; right hemisphere: medial frontal gyrus, superior frontal gyrus, and inferior temporal gyrus), poles (left temporal, right frontal and temporal gyrus), grooves (medial frontal right and left), and the right precuneus. In contrast, several cortical sulci of children with CH-Tx show a significant thickening with respect to those of the controls (left hemisphere: central, calcarine, pericalcarine and supramarginal sulcus; right hemisphere: medial orbitofrontal, medial occipital and superior occipital sulcus). Interestingly, the lower scores in the full-scale IQ of children with CH-Tx are associated with a thickening of the superior frontal, medial frontal, anterior cingulate, rectus, and orbital gyrus of the left hemisphere and the orbital gyrus of the right hemisphere [48]. At the functional level, some authors have consistently reported a decrease in intracortical conduction velocity [53,60,106], often evidenced by a higher latency of the P300 wave of the long latency-somatosensory evoked potential (LL-SEP) and a greater interpeak interval P40-P300 in children with CH-Tx [53,60]. These electrophysiological parameters negatively correlate with full-scale and performance IQ scores [53], suggesting that a poor performance in cognitive test execution could be partially explained by the neuroanatomical alterations in the gyrus of the brain cortex and a deficit in the intracortical conduction velocity.

Another study reported that school-age children and adolescents with CH-Tx (~9–15 years) showed a reduction in the volume of the hippocampus, specifically in the left hemisphere. In addition, a positive correlation was found between hippocampal volume and scores in memory tests, except in the Everyday Memory Functioning test [36]. Using functional MRI (fMRI), Wheeler et al. found that the hippocampus of adolescents with CH-Tx (12–15 years) presents an abnormal activation pattern. The hippocampus is activated bilaterally in the object-recognition test, unlike in control individuals where the activation occurs only in the left hippocampus. In addition, hypothyroid adolescents show areas of greater hippocampal activation versus controls in the object recognition (in both hemispheres) and recognition of novel spatial configurations (in the left hemisphere) tests; this occurs even without significant differences in the scores for those tests [16]. An abnormal activation pattern was also evident in adolescents with CH-Tx (12–15 years of age) during the processing of associative verbal memory. However, abnormal hippocampal activation is accompanied by deficits in various parameters of the memory test [61]. Overall, these findings support that perinatal THs deficiency leads to deficient hippocampal development in children with CH-Tx, which may include changes in neuronal circuits, resulting in modifications in the pattern and magnitude of hippocampal activation. The significance of these changes is still controversial because, in some cases, they could represent a compensatory mechanism that allows an optimal performance in some memory tasks (for example, in the visuospatial associative memory) [16]. In other cases, these changes could constitute a morphofunctional anomaly that explains the deficit in other types of memory (e.g., verbal associative memory) [61].

In line with the studies of memory, other studies have evaluated the cortical electrophysiological response during the processing of short-term recognition memory in children with CH-Tx aged 11–13 years. Although the study revealed an abnormal cortical response in hypothyroid children (lower amplitude of the P3 component of the ERPs), no significant changes were observed in the scores of the short-term recognition memory tests. This reinforces the notion that some electrophysiological changes manage to compensate for the behavioral response [17].

Functional alterations in other brain regions have been associated with deficits in visuospatial skills. School-age children with CH-Tx (aged 8–10 years) presented a different pattern of brain activation with respect to the control group during the mental rotation task. The control subjects showed activation of the superior parietal cortex, while children with CH showed greater activation in the bilateral supplementary motor area, opercular region of the precentral gyrus, adjacent insula, and the left somatosensory parietal cortex, along with lower activation of the inferior parietal cortex. It is suggested that a poor performance in the visuospatial domain of children with CH-Tx is particularly related to the decrease in the activation of the superior parietal cortex, which is necessary for the mental representation of the spatial characteristics of objects [59].

Alterations in the integrity of white matter in the brain are also linked to CH. Some case studies have shown pathological findings compatible with a demyelination process in pediatric patients with untreated CH; these alterations are reversed with L-T_4_ [107,108]. However, Cooper et al. described that the white matter of CH patients presents alterations that persist despite treatment. A multi-shell diffusion MRI (dMRI) showed that children with severe CH treated early presented microstructural alterations in the white matter, mainly in the cerebellum, thalamus, and right temporal lobe. Furthermore, there was a decrease in fractional anisotropy in the cerebellum, bilateral thalamus, and right temporal lobe; while increased radial diffusivity was detected in the cerebellum and bilateral thalamus; as well as in the increased transverse microscopic diffusivity was observed in the cerebellum, thalamus, occipital lobe, corpus callosum, and white matter adjacent to the sensorimotor cortex [52]. According to the authors, these findings suggest a reduction in the density of white matter fibers and an increase in their orientation and dispersion. It must be noted that the alterations in white matter were correlated with hearing, language, and communication deficits found in children with CH [52]. In these neuropsychological domains, conduction velocity also seems to play a key role. Marti et al. found higher latencies in the N200 component of the ERPs of patients with CH-Tx, which were negatively correlated with verbal IQ, indicating that the deficit in conduction velocity is associated with weak verbal skills [32].

In summary, the findings support that perinatal THs deficiency is enough to cause structural and electrophysiological changes in different subregions of the cerebral cortex and the hippocampus. In addition, it leads to modifications in the white matter of the cerebellum, the thalamus, the corpus callosum, and some cortical lobes. It is not possible to establish a cause-effect relationship between the morphofunctional alterations of the nervous system and the deficit in the neuropsychological domains of CH-Tx patients. Nevertheless, correlation studies provide valuable information that enables the investigation of the regions associated with the cognitive, sensory, and motor deficits. Above all, it provides an opportunity for researchers to design specific therapeutic strategies that mitigate the sequelae of CH.

### 4.5. Changes in Brain Thyroid Physiology: Thyroid Hormone Hyposensitivity

The effectiveness of treatment with L-T_4_ in patients with CH depends not only on providing the synthetic hormone at the appropriate dosage and time but also on other factors. Among them are THs transport from the periphery to the target organ, the metabolism of THs in situ, and the activation of their mechanism of action in the cell nucleus. All of them determine the effect of L-T_4_ both in the brain and the peripheral organs. When a failure occurs in any of the aforementioned factors, thyroid hormone hyposensitivity (THH) occurs. THH is defined as a state in which the biological activity of these hormones is affected by defects in their cellular transport, metabolism, or mechanism of action [109]. Some clinical findings suggest that patients with CH have a mild state of THH [110,111,112], which is manifested by alterations in the following parameters:

1. *Daily dose of L-T_4_.* Although there is still controversy [113], most reports have shown that pediatric CH patients require a higher daily dose of L-T_4_ than do patients with acquired or central hypothyroidism [111,114]. This has also been reported in young adults with this condition [110]. The increase in L-T_4_ dose suggests that tissues have a lower sensitivity to THs and there is a change in the set point of the hypothalamic–pituitary–thyroid axis, since higher doses are prescribed to counteract the clinical symptoms of hypothyroidism and maintain TSH levels within the reference range.
2. *Serum TSH levels.* When FT_4_ levels are within the reference range, 43% of patients with CH have hyperthyrotropinemia [111]. TSH is synthesized in the anterior pituitary gland, and its secretion is stimulated by a decrease in local T_3_ levels [115]. Therefore, the hyperthyrotropinemia observed in CH patients suggests a state of cerebral hypothyroidism that prevails despite peripheral euthyroidism.
3. *FT_3_/FT_4_ ratio*. Patients with CH exhibit a lower Free T_3_ (FT_3_)/FT_4_ ratio than those with acquired hypothyroidism [110]. This suggests a reduction in the efficiency of triiodothyronine (T_3_) synthesis from T_4_ at tissue level.
4. *TSH vs. FT_4_ curve.* Pediatric patients with CH show a rightward shift of the TSH/FT_4_ curve with respect to healthy individuals [112]. A similar change is observed in pediatric patients and young adults with CH when compared with patients with acquired hypothyroidism [110,111]. This indicates that a higher concentration of FT_4_ is required to maintain TSH within reference values, which supports the presence of a state of THH at the level of the central nervous system (CNS).

Clinical endocrine data raise the possibility that, unlike individuals whose thyroid deficiency occurs outside of the fetal stage (acquired hypothyroidism), CH patients present a state of THH in the CNS. THH in CH patients has three main characteristics. First, it occurs in only a percentage of patients with CH, as it has been reported in 43% of infants (<1 year of age) and 10% of children and adolescents (1–20 years of age) [112]. Second, THH is present in severe cases of CH, for example in patients with absence or total dysfunction of the thyroid gland [110,111]. Third, the severity of THH in CH patients is mild, as CH-Tx patients have FT_3_ and FT_4_ within the reference range, and normal or elevated TSH, as well as moderate neuropsychological alterations [88,110,111,112,114]. It should be noted that cases of severe THH are those associated with mutations or polymorphisms, and that unlike patients with mild THH, they frequently present intellectual disability and a thyroid profile with values outside the reference range, in addition to alterations in multiple systems (i.e., digestive, auditory, endocrine, cardiovascular, and muscular) [116].

**Figure 4:**
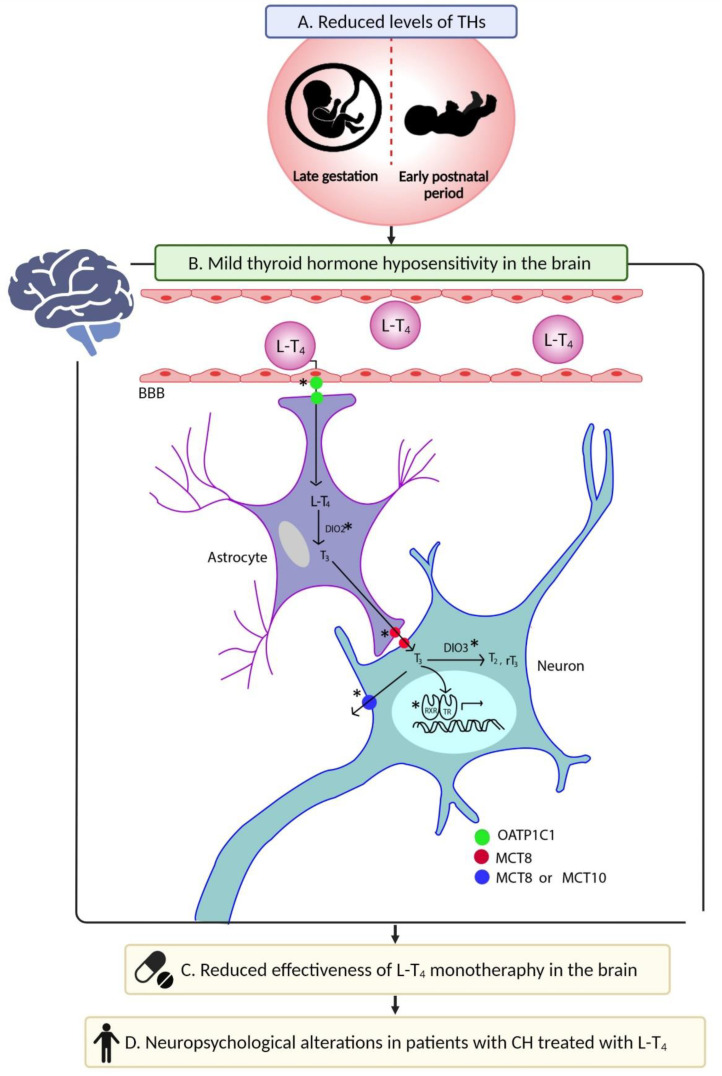
**Hypothesis of thyroid hormone hyposensitivity as a factor linked to neuropsychological alterations in patients with congenital hypothyroidism (CH) treated with levothyroxine (L-T_4_).** Reduced levels of thyroid hormones (THs) during perinatal period could lead to mild thyroid hormone hyposensitivity in the brain (**A**). Hyposensitivity may be caused by altered expression of molecules OATP1C, MCT8, DIO2, DIO3, and RXR and thyroid receptors (TRα1, TRβ1, TRβ2) (asterisks indicate molecules possibly affected). This leads to a deficit in L-T_4_ transport from the periphery to the brain, a reduced transport of L-T_4_ and T_3_ between neural cells, impaired conversion of L-T_4_ into T_3_ or T_3_ into T_2_, and an impaired THs mechanism of action in the brain (**B**). It is hypothesized that the hyposensitivity is mild but enough to reduce effectiveness of L-T_4_ treatment at cerebral level (**C**), which contributes to the incidence of neuropsychological alterations in CH patients under treatment (**D**). The mechanism of THs transport in this figure is based on that published by Alkemade et al. for human hypothalamus [118]. Abbreviations: BBB, Blood–brain barrier; OATP1CI, Organic anion-transporting polypeptide 1c1; MCT8, Monocarboxylate transporter 8; MCT10, Monocarboxylate transporter 10; DIO2, Type 2 deiodinase; DIO3, type 3 deiodinase; RXR, Retinoid X receptor; TR, thyroid receptors.

Although the evidence supports that THH in CH occurs in the hypothalamic–pituitary axis [110,111,112], this does not rule out the possibility that hyposensitivity is present in other brain regions, including those involved in cognitive, sensory and motor functions. To date, the molecular mechanism underlying THH in CH patients remains unknown. Still, genetic studies show that this condition is linked to alterations in different molecules, such as thyroid transporters, deiodinases, and thyroid receptors [109]. Under physiological conditions, these proteins participate in the supply of THs to the CNS and in their mechanism of action. Studies in cell lines [117] and human hypothalamic tissue [118,119,120] have allowed us to postulate a model of THs supply in which the interaction between astrocytes and neurons occurs. Briefly, T_4_ is transported to the cytoplasm of astrocytes through the organic anion transporter polypeptide 1C1 (OATP1C1). In the astrocytes, T_4_ is converted into T_3_ by the action of the enzyme deiodinase 2 (DIO2), and then T_3_ is taken out of the astrocyte by the monocarboxylate transporter 8 (MCT8). Finally, T_3_ enters neurons through MCT8, where it exerts its biological effect. Additionally, neuronal T_3_ can be converted into T_2_ by the action of the enzyme deiodinase 3 (DIO3) or exported from the neuron by the action of MCT8 or the monocarboxylate transporter 10 (MCT10) [118]. Regarding the mechanism of action of THs, it is known that T_3_, present in neurons, exerts its biological effect through a genomic mechanism involving the activation of thyroid receptors (TRα1, TRβ1, and TRβ2), the formation of heterodimers with retinoid X receptors (RXR), and gene transcription modulation (Figure 4B) [121].

Based on the molecular model described in the human hypothalamus [118], which is similar to that proposed for the brain of rodents [122], we hypothesized that the exposure to reduced levels of THs in the fetal period could potentially cause alterations in the expression of thyroid transporters, deiodinases, thyroid receptors or RXR. It may also lead to a deficient supply of L-T_4_ and a deficit in the mechanism of action of this hormone at the brain level and, consequently, a state of mild THH in the CNS of the patient with CH. To our knowledge, this hypothesis has not been explored at a molecular level in patients or animal models; however, previous research has shown a link between neonatal thyroid status and the sensitivity to THs at the brain level [123,124,125,126,127,128]. Srichomkwun et al. [125] demonstrated that the exposure to high concentrations of THs during intrauterine life causes brain THH in humans and mice. This effect is persistent until adulthood in both species and is accompanied by changes in the expression of deiodinases in the hypothalamus and pituitary. Interestingly, THH derived from intrauterine hyperthyroidism is persistent throughout three generations in the male human line [124] and is linked to an epigenetic phenomenon in the genes encoding the various molecules involved in the supply or mechanism of action of THs in the CNS [123].

Finally, the evidence supports that a proportion of CH patients have mild THH. Although the mechanism underlying this phenomenon is unknown, here we hypothesize that the exposure to reduced levels of THs during the perinatal period can lead to long-term changes in the expression of the molecules involved in the supply (OATP1C1 and MCT8), mechanism of action (TRα1, TRβ1, TRβ2, RXR), or metabolism (D2 and D3) of THs in the brain. These molecular changes could determine the effectiveness of treatment with L-T_4_ in patients with CH at the central level, thus contributing to the persistence of neuropsychological alterations reported in these patients (Figure 4). Clinical and basic research should be carried out to elucidate the molecular mechanism involved in the THH in CH and determine the contribution of this condition to the presence and/or severity of neuropsychological sequelae in patients with this disorder.

## 5. Research Agenda

- Study of CH-Tx patients with optimum neuropsychological profile and the factors linked to this satisfactory performance.
- Evaluation of the neuropsychological profile of adult and old-age patients with CH-Tx.
- Implementation of neuropsychological intervention therapies for patients with CH-Tx and evaluation of their therapeutic efficacy
- Search for structural and functional alterations in brain regions linked to language and sensorimotor, visuospatial and visuoconstructive abilities of patients with CH-Tx.
- Study of THH in patients with CH-Tx and its involvement in neuropsychological sequelae.

## 6. Conclusions

The evidence supports that patients with CH-Tx present neuropsychological sequelae throughout life. In the neonatal stage and childhood, alterations occur in the sensorimotor domain. In the preschool and school stages and adolescence, the six neuropsychological domains analyzed are affected, while a greater number of alterations are reported in school-age. In early adulthood, language seems to reach optimal levels, although IQ, mathematical skills, memory, and sensorimotor domains still show a deficit. The optimum L-T_4_ treatment is essential for mitigating the neuropsychological deficits, as adequate L-T_4_ doses and early time-to-treatment initiation allows CH patients to achieve IQ scores within reference values and prevent them from obtaining scores classified as low average, borderline, or extremely low.

Although the etiology of neuropsychological alterations in patients with CH-Tx is unknown, the literature shows that various extrinsic (social and related to L-T_4_ treatment) and intrinsic (severity and etiology of hypothyroidism, and anatomical and physiological brain alterations) factors interact to determine the establishment and severity of the sequelae. In addition, this review hypothesizes that THH could also contribute to the incidence of neuropsychological deficits in CH, as endocrinological data show the presence of a mild THH state at brain level in some patients with CH. This hypothesis proposes that exposure to reduced levels of THs during the perinatal period leads to a state of THH that is consolidated through changes in the expression of the molecules involved in the supply (OATP1C1 and MCT8), mechanism of action (TRα1, TRβ1, TRβ2, and RXR), or the metabolism (D2 and D3) of THs in the brain. These molecular changes could reduce the effectiveness of treatment with L-T_4_ in patients with CH-Tx at the central level, thus contributing to the persistence of neuropsychological alterations. Further research should be conducted to test this hypothesis and better characterize the neuropsychological alterations in patients with CH-Tx (in other age groups and their causes), as well as to implement and validate new psychological and endocrinological therapeutic strategies.

## Supplementary Materials

The following supporting information can be downloaded at: https://www.mdpi.com/article/10.3390/jcm11123427/s1, Table S1: Articles classified according to L-T_4_ treatment criteria published by the European Society for Pediatric Endocrinology and the European Society for Endocrinology [2], Table S2: Neuropsychological tests used in patients with CH-Tx, Table S3: Full-scale IQ scores in patients with CH-Tx.

## Abbreviations

CH	Congenital hypothyroidism
CH-Tx-	Congenital hypothyroidism treated with L-T_4_
CNS	Central nervous system
DIO2	Type 2 deiodinase
DIO3	Type 3 deiodinase
DUOX2	Dual oxidase 2
ERPs	Event related potentials
fMRI	Functional magnetic resonance imaging
FOXE1	Forkhead Box E1
FT_3_	Free T_3_
FT_4_	Free T_4_
IQ	Intellectual quotient
IYD	Iodotyrosine deiodinase
LL-SEP	Long latency somatosensory evoked potential
L-T_3_	Liothyronine
L-T_4_	Levothyroxine
MCT10	Monocarboxylate transporter 10
MCT8	Monocarboxylate transporter 8
MRI	Magnetic Resonance Imaging
msdMRI	Multi-Shell diffusion MRI
NKX2-1	NK2 homeobox 1
NKX2-5	NK2 homeobox 5
OATP1C1	Organic anion-transporting polypeptide 1c1
PAX8	Paired box 8
RXR	Retinoid X receptor
Scielo	Scientific Electronic Library Online
SLC26A4	Solute carrier family 26 member 4
SLC5A5	Solute carrier family 5 member 5
T_3_	Triiodothyronine
T_4_	Thyroxine
TG	Thyroglobulin
THH	Thyroid hormone hyposensitivity
THs	Thyroid hormones
TPO	Thyroid peroxidase
TRα1	Thyroid hormone receptor-α1
TRβ1	Thyroid hormone receptor β1
TRβ2	Thyroid hormone receptor β2
TSH	Thyrotropin
TSHR	Thyroid stimulating hormone receptor
