# Supplementary material for: Neuropsychological Alterations in Patients with Congenital Hypothyroidism Treated with Levothyroxine: Linked Factors and Thyroid Hormone Hyposensitivity
Source: J Clin Med. 2022 Jun 15;11(12):3427. doi: 10.3390/jcm11123427 (PMC9224966; doi:10.3390/jcm11123427)
Supplement: Supplementary file 1 [file jcm-11-03427-s001.zip › Table S1 and S2_V8.pdf]

Table S1. Articles classified according to L-T_4 treatment criteria published by the European Society for Pediatric Endocrinology and the European Society for Endocrinology [2]. Mean or median of the initial L-T_4 dose and starting time of treatment were considered for the classification of articles. Criteria for considering optimum L-T_4 treatment: 1) initial L-T_4 dose of 10-15 µg/kg/day or a lower dose (~10 µg/kg/day) for patients with mild CH (fT_4>10 pmol/L), 2) starting time of treatment no later than two weeks after birth (0-14 days), and 3) both parameters must be present. Highlighted in gray: articles reporting no neuropsychological alterations. The remaining articles (not highlighted) reported deficit in at least one neuropsychological domain.

Criteria	# of articles/total number of articles reviewed (%)	Articles
**A.** Articles reporting evidence from patients with an optimum L-T_4 treatment based on the criteria established in van Trotsenburg et al., 2021	9/69 (13.0)	-Bongers-Schokking et al., 2005 (early/high group)[1]<br>-Dimitropoulus et al., 2009[2]<br>-Ellerbroek et al., 2015[3]<br>-García et al., 2020[4]<br>-Marti et al., 2006[5]<br>-Rovet and Ehrlich et al.,1995 (LDG group)[6]<br>-Selva et al., 2005[7]<br>-Simic et al., 2013[8]<br>-Wheeler et al., 2011[9]
**B.** Articles reporting evidence from patients with a non-optimum L-T_4 treatment	35/69 (50.7)	-Álvarez et al., 1999[10]<br>-Alvarez et al., 2004[11]<br>-Arreóla-Ramírez et al., 2005[12]<br>-Bargagna et al., 1999[13]<br>-Bargagna et al., 2000[14]<br>-Blasi et al., 2009[15]<br>-Clairman et al., 2015[16]<br>-Corral-Guillé et al., 2019[17]<br>-Díaz-Pérez et al., 2018[18]<br>-Heyerdahl et al., 1991[19]<br>-Huo et al., 2011[20]<br>-Kempers et al., 2006[21]<br>-Kempers et al., 2007[22]<br>-Klein et al., 1972[23]<br>-Kooistra et al., 1994[24]<br>-Leneman et al., 2001[25]<br>-Oerbeck et al., 2003[26]<br>-Oerbeck et al., 2005[27]<br>-Olivares et al., 2004[28]<br>-Rachmiel et al., 2013[29]<br>-Rochiccioli et al., 1992[30]<br>-Rovet et al., 1992[31]<br>-Rovet, 1999[32]<br>-Rovet, 2005[33]<br>-Salerno et al., 1995[34]<br>-Salerno et al., 1999[35]<br>-Salerno et al., 2002[36]<br>-Seo et al., 2017[37]<br>-Soliman et al., 2012[38]<br>-Song et al., 2001[39]<br>-Weber et al., 1995[40]<br>-Weber et al., 2000[41]<br>-Wheeler et al., 2012[42]<br>-Wheeler et al., 2015[43]<br>-Willoughby et al., 2013[44]

**C**. Articles with imprecise data or no data on L-T_4 treatment	25/69 (36.2)	-Bongers-Schokking et al., 2016[45]<br>-Bongers-Schokking et al., 2018[46]<br>-Cooper et al., 2019[47]<br>-Dalili et al., 2014[48]<br>-Dassie-Leite et al., 2018[49]<br>-Frezzato et al., 2017[50]<br>-Gejao et al., 2009[51]<br>-Gleisner et al., 1986[52]<br>-Hepworth, et al. 2006[53]<br>-Herrera-Chinchay et al., 2021[54]<br>-J. Pediat, 1990, 116, 27–32[55]<br>-Komur et al., 2013[56]<br>-Lamônica et al., 2020[57]<br>-Mohamed et al., 2017[58]<br>-Najmi et al., 2013[59]<br>-Ordooei et al., 2014[60]<br>-Pardo et al., 2017[61]<br>-Pulungan et al., 2019[62]<br>-Rahmani et al., 2018[63]<br>-Ramírez and Marchena, 2009[64]<br>-Rivera-González et al., 2014[65]<br>-Romero et al., 2011[66]<br>-Simons et al., 1997[67]<br>-Soliman et al., 2016[68]<br>-Ulloa et al., 2016[69]

References

1. Bongers-Schokking, J.J.; de Muinck Keizer-Schrama, S.M.P.F. Influence of Timing and Dose of Thyroid Hormone Replacement on Mental, Psychomotor, and Behavioral Development in Children with Congenital Hypothyroidism. *The Journal of pediatrics* **2005**, *147*, 768–774, doi:10.1016/j.jpeds.2005.09.031.

2. Dimitropoulos, A.; Molinari, L.; Etter, K.; Torresani, T.; Lang-Muritano, M.; Jenni, O.G.; Largo, R.H.; Latal, B. Children with Congenital Hypothyroidism: Long-Term Intellectual Outcome after Early High-Dose Treatment. *Pediatr Res* **2009**, *65*, 242–248, doi:10.1203/PDR.0b013e31818d2030.

3. Ellerbroek, V.L.; Bonfig, W.; Dörr, H.-G.; Bettendorf, M.; Hauffa, B.; Fricke-Otto, S.; Rohrer, T.; Reschke, F.; Schönau, E.; Schwab, K.O.; et al. Long-Term Surveillance of Children with Congenital Hypothyroidism: Data from the German Registry for Congenital Hypothyroidism (AQUAPE "Hypo Dok"). *Klinische Padiatrie* **2015**, *227*, 199–205, doi:10.1055/s-0035-1549978.

4. García Morales, L.; Rodríguez Arnao, M.D.; Rodríguez Sánchez, A.; Dulín Íñiguez, E.; Álvarez González, M.A. Sustained Attention in School-Age Children with Congenital Hypothyroidism: Influence of Episodes of Overtreatment in the First Three Years of Life. *Neurologia (Barcelona, Spain)* **2020**, *35*, 226–232, doi:10.1016/j.nrl.2017.08.003.

5. Marti, S.; Alvarez, M.; Simoneau-Roy, J.; Leroux, S.; Van Vliet, G.; Robaey, P. Effects of Early High-Dose Levothyroxine Treatment on Auditory Brain Event-Related Potentials at School Entry in Children with Congenital Hypothyroidism. *Hormone research* **2006**, *66*, 240–248, doi:10.1159/000095069.

6. Rovet, J.F.; Ehrlich, R.M. Long-Term Effects of L-Thyroxine Therapy for Congenital Hypothyroidism. *The Journal of pediatrics* **1995**, *126*, 380–386, doi:10.1016/s0022-3476(95)70452-3.

7. Selva, K.A.; Harper, A.; Downs, A.; Blasco, P.A.; Lafranchi, S.H. Neurodevelopmental Outcomes in Congenital Hypothyroidism: Comparison of Initial T4 Dose and Time to Reach Target T4 and TSH. *The Journal of pediatrics* **2005**, *147*, 775–780, doi:10.1016/j.jpeds.2005.07.024.

8. Simic, N.; Khan, S.; Rovet, J. Visuospatial, Visuoperceptual, and Visuoconstructive Abilities in Congenital Hypothyroidism. *Journal of the International Neuropsychological Society : JINS* **2013**, *19*, 1119–1127, doi:10.1017/S1355617713001136.

9. Wheeler, S.M.; Willoughby, K.A.; McAndrews, M.P.; Rovet, J.F. Hippocampal Size and Memory Functioning in Children and Adolescents with Congenital Hypothyroidism. *The Journal of clinical endocrinology and metabolism* **2011**, *96*, E1427-34, doi:10.1210/jc.2011-0119.

10. Álvarez MA, Güell R, Daniel L, Reyes-Berazaín A, Machado C, P.A. Estado Neurocognitivo En Niños de 8 Años Con Hipotiroidismo Congénito Tratado Precozmentetle. *Rev Neurol* **1999**, *28*, 701–706.

11. Alvarez, M.; Carvajal, F.; Renón, A.; Pérez, C.; Olivares, A.; Rodríguez, G.; Alvarez, V. Differential Effect of Fetal, Neonatal and Treatment Variables on Neurodevelopment in Infants with Congenital Hypothyroidism. *Hormone research* **2004**, *61*, 17–20, doi:10.1159/000075192.

12. Arreóla-Ramírez, G.; Barrera-Reyes, R.H.; Jiménez-Quiroz, R.; Ramírez Torres, M.A.; Segura-Cervantes, E.; Granados-Cepeda, M.L.; Ramírez-Vargas, M.N.; Meza-Rodríguez, M. del P. Neurodesarrollo En Infantes Con Antecedente de Hipotiroidismo Congénito . *Perinatología y reproducción humana* 2005, *19*, 141–151.

13. Bargagna, S.; Dinetti, D.; Pinchera, A.; Marcheschi, M.; Montanelli, L.; Presciuttini, S.; Chiovato, L. School Attainments in Children with Congenital Hypothyroidism Detected by Neonatal Screening and Treated Early in Life. *European journal of endocrinology* **1999**, *140*, 407–413, doi:10.1530/eje.0.1400407.

14. Bargagna, S.; Canepa, G.; Costagli, C.; Dinetti, D.; Marcheschi, M.; Millepiedi, S.; Montanelli, L.; Pinchera, A.; Chiovato, L. Neuropsychological Follow-up in Early-Treated Congenital Hypothyroidism: A Problem-Oriented Approach. *Thyroid* **2000**, *10*, 243–249, doi:10.1089/thy.2000.10.243.

15. Blasi, V.; Longaretti, R.; Giovanettoni, C.; Baldoli, C.; Pontesilli, S.; Vigone, C.; Saccuman, C.; Nigro, F.; Chiumello, G.; Scotti, G.; et al. Decreased Parietal Cortex Activity during Mental Rotation in Children with Congenital Hypothyroidism. *Neuroendocrinology* **2009**, *89*, 56–65, doi:10.1159/000151397.

16. Clairman, H.; Skocic, J.; Lischinsky, J.E.; Rovet, J. Do Children with Congenital Hypothyroidism Exhibit Abnormal Cortical Morphology? *Pediatric research* **2015**, *78*, 286–297, doi:10.1038/pr.2015.93.

17. Corral-Guillé, I.; Rivera-González, R.; Ontiveros-Mendoza, E. Psychomotricity and Its Relationship with the Intelligence Quotient in Preschoolers with Congenital Hypothyroidism. *Int Phys Med Rehab J* **2019**, *4*, 70–76.

18. Díaz Pérez, E.J.; Sánchez Pérez, M. del C.; Moreno Macías, H.; Echeverría Arjonilla, J.C.; Rivera González, I.R.; Calzada León, R.; Ruíz-Reyes, M. de la L.; Ontiveros Mendoza, E.; Nelly, B.-A.; Antonio, M.V.M. Hipotiroidismo Congénito Primario y Neurodesarrollo: Un Enfoque Terapéutico Integral. *Acta Pediátrica de México* **2018**, *39*, 299–315.

19. Heyerdahl, S.; Kase, B.F.; Lie, S.O. Intellectual Development in Children with Congenital Hypothyroidism in Relation to Recommended Thyroxine Treatment. *The Journal of pediatrics* **1991**, *118*, 850–857, doi:10.1016/s0022-3476(05)82194-8.

20. Huo, K.; Zhang, Z.; Zhao, D.; Li, H.; Wang, J.; Wang, X.; Feng, H.; Wang, X.; Zhu, C. Risk Factors for Neurodevelopmental Deficits in Congenital Hypothyroidism after Early Substitution Treatment. *Endocr J* **2011**, *58*, 355–361, doi:10.1507/endocrj.k10e-384.

21. Kempers, M.J.E.; van der Sluijs Veer, L.; Nijhuis-van der Sanden, M.W.G.; Kooistra, L.; Wiedijk, B.M.; Faber, I.; Last, B.F.; de Vijlder, J.J.M.; Grootenhuis, M.A.; Vulsma, T. Intellectual and Motor Development of Young Adults with Congenital Hypothyroidism Diagnosed by Neonatal Screening. *The Journal of clinical endocrinology and metabolism* **2006**, *91*, 418–424, doi:10.1210/jc.2005-1209.

22. Kempers, M.J.E.; van der Sluijs Veer, L.; Nijhuis-van der Sanden, R.W.G.; Lanting, C.I.; Kooistra, L.; Wiedijk, B.M.; Last, B.F.; de Vijlder, J.J.M.; Grootenhuis, M.A.; Vulsma, T. Neonatal Screening for Congenital Hypothyroidism in the Netherlands: Cognitive and Motor Outcome at 10 Years of Age. *The Journal of clinical endocrinology and metabolism* **2007**, *92*, 919–924, doi:10.1210/jc.2006-1538.

23. Klein, A.H.; Meltzer, S.; Kenny, F.M. Improved Prognosis in Congenital Hypothyroidism Treated before Age Three Months. *J Pediatr* **1972**, *81*, 912–915, doi:10.1016/s0022-3476(72)80542-0.

24. Kooistra, L.; Laane, C.; Vulsma, T.; Schellekens, J.M.; van der Meere, J.J.; Kalverboer, A.F. Motor and Cognitive Development in Children with Congenital Hypothyroidism: A Long-Term Evaluation of the Effects of Neonatal Treatment. *J Pediatr* **1994**, *124*, 903–909, doi:10.1016/s0022-3476(05)83178-6.

25. Leneman, M.; Buchanan, L.; Rovet, J. Where and What Visuospatial Processing in Adolescents with Congenital Hypothyroidism. *J Int Neuropsychol Soc* **2001**, *7*, 556–562, doi:10.1017/s1355617701755038.

26. Oerbeck, B.; Sundet, K.; Kase, B.F.; Heyerdahl, S. Congenital Hypothyroidism: Influence of Disease Severity and L-Thyroxine Treatment on Intellectual, Motor, and School-Associated Outcomes in Young Adults. *Pediatrics* **2003**, *112*, 923–930, doi:10.1542/peds.112.4.923.

27. Oerbeck, B.; Sundet, K.; Kase, B.F.; Heyerdahl, S. Congenital Hypothyroidism: No Adverse Effects of High Dose Thyroxine Treatment on Adult Memory, Attention, and Behaviour. *Archives of Disease in Childhood* **2005**, *90*, 132 LP – 137, doi:10.1136/adc.2003.043935.

28. Olivares Torres, A.; Carlos Pías, N.; Mar Rodríguez, C.; Pérez Gesen, C.; Carvajal Martínez, F.; Rojas, E.; Acosta, C.; Fernández Yero, J.L.; Álvarez González, M.Á. Atención Sostenida En Niños En Edad Escolar Con Hipotiroidismo Congénito. *Revista Cubana de Endocrinología* **2004**, *15*, 0.

29. Rachmiel, M.; Blaser, S.; Widjaja, E.; Rovet, J. Children with Congenital Hypothyroidism Have Similar Neuroradiological Abnormal Findings as Healthy Ones. *ScientificWorldJournal* **2013**, *2013*, 194918, doi:10.1155/2013/194918.

30. Rochiccioli, P.; Roge, B.; Alexandre, F.; Tauber, M.T. School Achievement in Children with Hypothyroidism Detected at Birth and Search for Predictive Factors. *Hormone Research in Paediatrics* **1992**, *38*, 236–240.

31. Rovet, J.F.; Ehrlich, R.M.; Sorbara, D.L. Neurodevelopment in Infants and Preschool Children with Congenital Hypothyroidism: Etiological and Treatment Factors Affecting Outcome. *J Pediatr Psychol* **1992**, *17*, 187–213, doi:10.1093/jpepsy/17.2.187.

32. Rovet, J.F. Long-Term Neuropsychological Sequelae of Early-Treated Congenital Hypothyroidism: Effects in Adolescence. *Acta Paediatr Suppl* **1999**, *88*, 88–95, doi:10.1111/j.1651-2227.1999.tb01168.x.

33. Rovet, J.F. Children with Congenital Hypothyroidism and Their Siblings: Do They Really Differ? *Pediatrics* **2005**, *115*, e52-7, doi:10.1542/peds.2004-1492.

34. Salerno, M.; di Maio, S.; Militerni, R.; Argenziano, A.; Valerio, G.; Tenore, A. Prognostic Factors in the Intellectual Development at 7 Years of Age in Children with Congenital Hypothyroidism. *J Endocrinol Invest* **1995**, *18*, 774–779, doi:10.1007/BF03349810.

35. Salerno, M.; Militerni, R.; di Maio, S.; Bravaccio, C.; Gasparini, N.; Tenore, A. Intellectual Outcome at 12 Years of Age in Congenital Hypothyroidism. *Eur J Endocrinol* **1999**, *141*, 105–110, doi:10.1530/eje.0.1410105.

36. Salerno, M.; Militerni, R.; Bravaccio, C.; Micillo, M.; Capalbo, D.; Di, M.S.; Tenore, A. Effect of Different Starting Doses of Levothyroxine on Growth and Intellectual Outcome at Four Years of Age in Congenital Hypothyroidism. *Thyroid* **2002**, *12*, 45–52, doi:10.1089/105072502753451968.

37. Seo, M.K.; Yoon, J.S.; So, C.H.; Lee, H.S.; Hwang, J.S. Intellectual Development in Preschool Children with Early Treated Congenital Hypothyroidism. *Ann Pediatr Endocrinol Metab* **2017**, *22*, 102–107, doi:10.6065/apem.2017.22.2.102.

38. Soliman, A.T.; Azzam, S.; Elawwa, A.; Saleem, W.; Sabt, A. Linear Growth and Neurodevelopmental Outcome of Children with Congenital Hypothyroidism Detected by Neonatal Screening: A Controlled Study. *Indian J Endocrinol Metab* **2012**, *16*, 565–568, doi:10.4103/2230-8210.98012.

39. Song, S.I.; Daneman, D.; Rovet, J. The Influence of Etiology and Treatment Factors on Intellectual Outcome in Congenital Hypothyroidism. *J Dev Behav Pediatr* **2001**, *22*, 376–384, doi:10.1097/00004703-200112000-00005.

40. Weber, G.; Siragusa, V.; Rondanini, G.F.; Prina Cerai, L.M.; Mora, S.; Colombini, J.; Medaglini, S.; Lia, C.; Locatelli, T.; Comi, G. Neurophysiologic Studies and Cognitive Function in Congenital Hypothyroid Children. *Pediatr Res* **1995**, *37*, 736–740, doi:10.1203/00006450-199506000-00011.

41. Weber, G.; Mora, S.; Prina Cerai, L.M.; Siragusa, V.; Colombini, J.; Medaglini, S.; Fornara, C.; Locatelli, T.; Comi, G.; Chiumello, G. Cognitive Function and Neurophysiological Evaluation in Early-Treated Hypothyroid Children. *Neurological sciences : official journal of the Italian Neurological Society and of the Italian Society of Clinical Neurophysiology* **2000**, *21*, 307–314, doi:10.1007/s100720070068.

42. Wheeler, S.M.; McAndrews, M.P.; Sheard, E.D.; Rovet, J. Visuospatial Associative Memory and Hippocampal Functioning in Congenital Hypothyroidism. *J Int Neuropsychol Soc* **2012**, *18*, 49–56, doi:10.1017/S1355617711001378.

43. Wheeler, S.M.; McLelland, V.C.; Sheard, E.; McAndrews, M.P.; Rovet, J.F. Hippocampal Functioning and Verbal Associative Memory in Adolescents with Congenital Hypothyroidism. *Front Endocrinol (Lausanne)* **2015**, *6*, 163, doi:10.3389/fendo.2015.00163.

44. Willoughby, K.A.; McAndrews, M.P.; Rovet, J. Effects of Early Thyroid Hormone Deficiency on Children's Autobiographical Memory Performance. *J Int Neuropsychol Soc* **2013**, *19*, 419–429, doi:10.1017/S1355617712001488.

45. Bongers-Schokking, J.J.; Resing, W.C.M.; Oostdijk, W.; de Rijke, Y.B.; de Muinck Keizer-Schrama, S.M.P.F. Individualized Treatment to Optimize Eventual Cognitive Outcome in Congenital Hypothyroidism. *Pediatr Res* **2016**, *80*, 816–823, doi:10.1038/pr.2016.159.

46. Bongers-Schokking, J.J.; Resing, W.C.M.; Oostdijk, W.; de Rijke, Y.B.; de Muinck Keizer-Schrama, S.M.P.F. Relation between Early Over- and Undertreatment and Behavioural Problems in Preadolescent Children with Congenital Hypothyroidism. *Hormone research in paediatrics* **2018**, *90*, 247–256, doi:10.1159/000494056.

47. Cooper, H.E.; Kaden, E.; Halliday, L.F.; Bamiou, D.-E.; Mankad, K.; Peters, C.; Clark, C.A. White Matter Microstructural Abnormalities in Children with Severe Congenital Hypothyroidism. *Neuroimage Clin* **2019**, *24*, 101980, doi:10.1016/j.nicl.2019.101980.

48. Dalili, S.; Rezvani, S.M.; Dalili, H.; Mohtasham Amiri, Z.; Mohammadi, H.; Abrisham Kesh, S.; Novin, M.H.; Medghalchi, A.; Gholamnezhad, H. Congenital Hypothyroidism: Etiology and Growth-Development Outcome. *Acta Med Iran* **2014**, *52*, 752–756.

49. Dassie-Leite, A.P.; Behlau, M.; Nesi-França, S.; Lima, M.N.; Lacerda, L. de Phonological Acquisition in Children with Early-Treated Congenital Hypothyroidism: Association with Clinical and Laboratory Parameters. *Codas* **2018**, *30*, e20180013, doi:10.1590/2317-1782/20182018013.

50. Frezzato, R.C.; Santos, D.C.C.; Goto, M.M.F.; Ouro, M.P.C. do; Santos, C.T.M. dos; Dutra, V.; Lima, M.C.M.P. Fine motor skills and expressive language: a study with children with congenital hypotyreoidism. *Codas* **2017**, *29*, e20160064, doi:10.1590/2317-1782/20172016064.

51. Gejão, M.G.; Ferreira, A.T.; Silva, G.K.; Anastácio-Pessan, F. da L.; Lamônica, D.A.C. Communicative and Psycholinguistic Abilities in Children with Phenylketonuria and Congenital Hypothyroidism. *J Appl Oral Sci* **2009**, *17 Suppl*, 69–75, doi:10.1590/s1678-77572009000700012.

52. Gleisner, A.; Torres, C.; Wilhelm, V.; Asenjo, S.; Adriazola, A.; Cafati, I.; Valenzuela, E.; Lillo, R. [Congenital hypothyroidism: neurological and psychometric evaluation]. *Revista chilena de pediatria* **1986**, *57*, 524–527.

53. Hepworth, S.L.; Pang, E.W.; Rovet, J.F. Word and Face Recognition in Children with Congenital Hypothyroidism: An Event-Related Potential Study. *J Clin Exp Neuropsychol* **2006**, *28*, 509–527, doi:10.1080/13803390590949331.

54. Herrera-Chinchay, L.; Silva-Ocas, I.; Castro-Silva, N.; del Águila Villar, C. [Social, cognitive and psychomotor development in peruvian children with congenital hypothyroidism]. *Andes pediatrica : revista Chilena de pediatria* **2021**, *92*, 235–240, doi:10.32641/andespediatr.v92i2.1955.

55. Elementary School Performance of Children with Congenital Hypothyroidism. New England Congenital Hypothyroidism Collaborative. *J Pediatr* **1990**, *116*, 27–32, doi:10.1016/s0022-3476(05)81640-3.

56. Komur, M.; Ozen, S.; Okuyaz, C.; Makharoblidze, K.; Erdogan, S. Neurodevelopment Evaluation in Children with Congenital Hypothyroidism by Bayley-III. *Brain Dev* **2013**, *35*, 392–397, doi:10.1016/j.braindev.2012.07.003.

57. Lamônica, D.A.C.; Anastácio-Pessan, F. da L.; Ferraz, P.M.D.P.; Ribeiro, C. da C. Performance in motor, communicative and cognitive skills of girls with congenital hypothyroidism treated from the neonatal period. *Codas* **2020**, *32*, e20190017, doi:10.1590/2317-1782/20192019017.

58. Mohamed, H.M.; Zaki, E.; Abdall, A.A.B.; Gomaa, M.A.; Wahab, M.M.A. Language Disorders in Children with Congenital Hypothyroidism. *The Egyptian Journal of Otolaryngology* **2017**, *33*, 685–690.

59. Najmi, S.B.; Hashemipour, M.; Maracy, M.R.; Hovsepian, S.; Ghasemi, M. Intelligence Quotient in Children with Congenital Hypothyroidism: The Effect of Diagnostic and Treatment Variables. *J Res Med Sci* **2013**, *18*, 395–399.

60. Ordooei, M.; Mottaghipisheh, H.; Fallah, R.; Rabiee, A. Cognitive Outcomes for Congenital Hypothyroid and Healthy Children: A Comparative Study. *Iran J Child Neurol* **2014**, *8*, 28–32.

61. Pardo Campos, M.L.; Musso, M.; Keselman, A.; Gruñeiro, L.; Bergadá, I.; Chiesa, A. Cognitive Profiles of Patients with Early Detected and Treated Congenital Hypothyroidism. *Archivos argentinos de pediatria* **2017**, *115*, 12–17, doi:10.5546/aap.2017.eng.12.

62. Pulungan, A.B.; Oldenkamp, M.E.; Trotsenburg, A.S.P. van; Windarti, W.; Gunardi, H. Effect of Delayed Diagnosis and Treatment of Congenital Hypothyroidism on Intelligence and Quality of Life: An Observational Study. *Medical Journal of Indonesia* **2019**, *28*, doi:10.13181/mji.v28i4.3473.

63. Rahmani, K.; Yarahmadi, S.; Etemad, K.; Mehrabi, Y.; Aghang, N.; Koosha, A.; Soori, H. Intelligence Quotient at the Age of Six Years of Iranian Children with Congenital Hypothyroidism. *Indian Pediatr* **2018**, *55*, 121–124.

64. Ramírez, Y.; Marchena, H. Características Neuropsicológicas Del Niño Preescolar Con Hipotiroidismo Congénito En La Provincia de Cienfuegos. *Revista chilena de Neuropsicología* **2009**, *4*, 36–43.

65. Rivera González, R.; Figueroa Olea, M.; Soto Romas, C.; Soto Villaseñor, F.; Sánchez, C. Interacción Madre-Hijo Durante La Alimentación En Niños Con Hipotiroidismo Congénito. *Revista de Enfermería Neurológica* **2014**, *13*, 64–69.

66. Romero, J.B.; Palacios, G.C.; Gómez, N.; Silva, A.; Fabela, J.H. [Intelligence quotient related with congenital hypotyroidism etiology]. *Revista medica del Instituto Mexicano del Seguro Social* **2011**, *49*, 179–183.

67. Simons, W.F.; Fuggle, P.W.; Grant, D.B.; Smith, I. Educational Progress, Behaviour, and Motor Skills at 10 Years in Early Treated Congenital Hypothyroidism. *Arch Dis Child* **1997**, *77*, 219–222, doi:10.1136/adc.77.3.219.

68. Soliman, H.; Abdel Hady, A.F.; Abdel Hamid, A.; Mahmoud, H. Language Profile in Congenital Hypothyroid Children Receiving Replacement Therapy. *Folia phoniatrica et logopaedica : official organ of the International Association of Logopedics and Phoniatrics (IALP)* **2016**, *68*, 67–72, doi:10.1159/000448744.

69. Ulloa, B.E.C.; Saquipay, Á.D.C.; Matute, J.A.H.; Castro, J.E.C.; Brito, M.C.P. Neurodesarrollo En El Hipotiroidismo Congénito y Sus Particularidades Electroencefalográficas. *Revista de la Facultad de Ciencias Médicas de la Universidad de Cuenca* **2016**, *34*, 41–46.

Table S2. Neuropsychological tests used in patients with CH-Tx. This table was based on Núñez et al[1].

**1. Intellectual quotient**
**Wechsler Abbreviated Scale of Intelligence (WASI)**<br>**Stanford-Binet Intelligence Scale (SBIS)**<br>**Wechsler Intelligence Scale for Children, third edition (WISC-III)**<br>**Wechsler Preschool and Primary Scales of Intelligence (WPPSI)**<br>**Short version of the Revised Amsterdam Child Intelligence**<br>**Castell Test of Infant Intelligence**<br>**Dutch version of the Wechsler Adult Intelligence Scale**<br>**Korean Wechsler Intelligence Scale**<br>**Revised Amsterdam Child Intelligence Test (RAKIT)**<br>**Wechsler Intelligence Scale for Children 4th Edition (WISC-IV)**<br>**Wechsler Intelligence Test for Children (WISC-R)**<br>**Wechsler-Bellevue Intelligence**<br>**Griffiths Mental Development Scale**
**2. Language**
**Carrow Language Scales**<br>**Wepman's Auditory Discrimination Test**<br>**Peabody Picture Vocabulary Test - Revised (PPVT-R)**<br>**ABFW Child Language Test**<br>**Clinical Linguistic and Auditory Milestone Scale**<br>**Early Language Milestone Scale**<br>**Language Development Evaluation Scale**<br>**ABFW Child Language Test-Phonology**<br>**Illinois Test of Psycholinguistic Abilities**<br>**Language Fundamentals UK**<br>**Verbal Fluency Test**<br>**Children's Auditory Verbal Learning Test (CAVLT)**
**3. Learning**
**Neal Analysis of Reading Ability**<br>**School achievement Tests (Bristol)**<br>**Norwegian Observational Test of Reading and Writing**<br>**School Achievement Tests**<br>**School Maturity Test**<br>**Wisconsin Card Sorting Test (WCST)**<br>**WRAT3 (Wide Range Achievement Test)**<br>**VPS-R Test of Visual-Perceptual Skills Revised**
**4. Memory**
**Children's Memory Scale Stories**<br>**Rey-Osterrieth Complex Figure Test (REY-O)**<br>**Everyday Memory Questionnaire**<br>**Children's Autobiographical Interview (CAI)**<br>**Continuous Visual Memory Test (CVMT)**<br>**WMS-R Logical Memory (WMS-R-LM)**
**5. Motor area**

**The Bender Visual Motor Gestalt Test**
**The Beery Developmental Test of Visual Motor Integration**
**Movement Assessment Battery for Children (MABC)**
**Corsi Block-Tapping Test**
**Brunet-Lézine Maturity Scale**
**Global Development**
**McCarthy Scales of Children's Abilities**
**NEPSY**
**Denver Developmental Screening Test, 2nd edition (DDST-II)**
**The Bayley Scales of Infant and Toddler Development III- Screening test BSITD III**
**Gesell Developmental Schedules (GDS)**
**Initial Luria Battery**

References

1. Núñez, A.; Bedregal, P.; Becerra, C.; Grob, F. Alteraciones Del Neurodesarrollo En Pacientes Con Hipotiroidismo Congénito: Recomendaciones Para El Seguimiento. *Rev. Médica Chile* **2017**, *145*, 1579–1587.
